# Preoperative prediction of the need for postoperative adjuvant therapy in stage IB cervical cancer using tumor size measured on magnetic resonance imaging and serum squamous cell carcinoma antigen levels

**DOI:** 10.1016/j.xagr.2026.100671

**Published:** 2026-06-28

**Authors:** Michihide Maeda, Miho Kitai, Masashi Akada, Yusaku Shimizu, Eri Yamabe, Reisa Kakubari, Shinya Matsuzaki, Tsuyoshi Hisa, Shinsuke Koyama, Seiji Mabuchi

**Affiliations:** 1 Department of Gynecology, Osaka International Cancer Institute, Osaka, Japan (Maeda, Kitai, Akada, Shimizu, Yamabe, Kakubari, Matsuzaki, Hisa, Mabuchi); 2 Department of Obstetrics and Gynecology, Osaka Police Hospital, Osaka, Japan (Maeda and Koyama); 3 Department of Obstetrics and Gynecology, The University of Osaka, Suita, Osaka, Japan (Akada, Shimizu, Matsuzaki); 4 Department of Obstetrics and Gynecology, Hyogo Medical University, Nishinomiya, Hyogo, Japan (Mabuchi)

**Keywords:** adjuvant therapy, cervical cancer, magnetic resonance imaging, treatment de-escalation, tumor marker

## Abstract

**OBJECTIVE:**

Radical hysterectomy is widely performed for stage IB cervical cancer; however, a substantial number of patients require postoperative adjuvant therapy, resulting in dual-modality treatment. This study evaluated the predictive value of tumor size measured on magnetic resonance imaging (MRI) and serum squamous cell carcinoma (SCC) antigen levels for the need for postoperative adjuvant therapy.

**METHODS:**

We retrospectively analyzed 191 patients with stage IB cervical SCC who underwent radical hysterectomy. Patients were also analyzed in a stage IB1 to IB2 cohort. The cut-off values of preoperative tumor size measured on MRI and serum SCC antigen levels for the need for postoperative adjuvant therapy were determined using receiver operating characteristic curve analysis.

**RESULTS:**

A total of 104 patients (54.5%) received postoperative adjuvant therapy. Large tumor size measured on MRI and elevated serum SCC antigen levels were predictors of the need for postoperative adjuvant therapy. The optimal cut-off values were 3.2 cm and 2.3 ng/mL in the overall cohort, and 2.4 cm and 2.3 ng/mL in the stage IB1 to IB2 cohort. Patients were stratified into three risk groups according to the number of predictive factors. In the low-risk group (no predictors), 20 of 86 patients (23.3%) required postoperative adjuvant therapy. In the intermediate-risk group (one predictor), 46 of 64 patients (71.9%) required adjuvant therapy. In the high-risk group (two predictors), 38 of 41 patients (92.7%) received adjuvant therapy. Similar trends were observed in the stage IB1 to IB2 cohort, with increasing rates of adjuvant therapy across risk groups.

**CONCLUSION:**

Preoperative tumor size measured on MRI and serum SCC antigen levels are useful predictors of the need for postoperative adjuvant therapy in stage IB cervical SCC. Their combination could assist in treatment planning and patient counseling.

**AJOG Global Reports at a Glance**

**Why was this study conducted?**

This study evaluated the predictive value of tumor size measured on magnetic resonance imaging (MRI) and serum squamous cell carcinoma (SCC) antigen levels for the need for postoperative adjuvant therapy in patients with stage IB cervical squamous cell carcinoma.

**What are the key findings?**

Patients with large tumor size measured on MRI and elevated serum SCC levels are more likely to require postoperative adjuvant therapy.

**What does this study add to what is already known?**

These findings highlight the potential clinical utility of incorporating tumor size measured on MRI and serum SCC antigen levels into preoperative decision-making for early-stage cervical squamous cell carcinoma.

## Introduction

Cervical cancer is the fourth most common malignancy among women worldwide.^1^ For early-stage cervical cancer (stage IB–IIA), radical hysterectomy or chemoradiotherapy (CRT) was performed. In surgically treated patients, adjuvant therapy is required according to the postoperative recurrence risk.^2^^,^^3^

Although surgery has some advantages, particularly in preserving ovarian function and reducing bowel dysfunction,^4^ the addition of adjuvant therapy may increase treatment-related morbidity without clear survival benefit in certain settings.^5^, ^6^, ^7^, ^8^ Therefore, an accurate preoperative assessment of recurrence risk, and consequently the need for adjuvant therapy, is important for planning treatment and improving the post-treatment quality of life. Tumor size measured on magnetic resonance imaging (MRI) and preoperative serum squamous cell carcinoma (SCC) antigen levels are commonly used in clinical practice; they have shown potential as predictive markers of pathological recurrence risk. However, the use of these two factors and their combinations to determine the need for adjuvant therapy has not yet been fully evaluated.

In this study, we retrospectively investigated the clinical utility of tumor size measured on MRI and serum SCC antigen levels to predict the need for adjuvant therapy in patients with stage IB cervical SCC.

## Materials and methods

### Study design and patient eligibility

This single-center retrospective study was approved by the Institutional Review Board of Osaka International Cancer Institute (No. 25099). Informed consent was obtained from all participants. Patients with cervical cancer who underwent type C1 radical hysterectomy (Querleu–Morrow classification) between January 2010 and December 2020 were identified from our institutional database. Patients who met the following criteria were identified: (1) histologically confirmed SCC, (2) stage IB disease according to the 2018 International Federation of Gynecology and Obstetrics (FIGO) staging system, (3) preoperative measurement of serum SCC antigen levels, and (4) preoperative pelvic MRI performed before cervical conization. Patients with preoperatively diagnosed parametrial invasion, lymph node metastasis, or synchronous malignancies were excluded. Because the FIGO staging system was revised during the study period (2010–2020), all patients were retrospectively restaged according to the 2018 FIGO staging system.

Postoperative adjuvant therapy was administered based on the pathological risk factors. High risk was defined according to the Peters criteria, and intermediate risk was defined according to the Sedlis criteria.^9^^,^^10^ Treatment decision was made through a multidisciplinary tumor board discussion involving gynecologic oncologists, radiation oncologists, and pathologists.

### Clinical variables

Clinical variables, including patient demographics, FIGO stage, tumor size measured on MRI, and preoperative serum SCC antigen levels, were extracted from the institutional database.

### Definition of tumor size, parametrial invasion, and pelvic lymph node metastasis

Tumor size was evaluated using T2-weighted MRI. The length, width, and height of the lesions were measured, and the longest diameter was defined as the tumor size. Preoperative parametrial invasion was defined as disruption of the stromal ring or tumor extension into the parametrium on T2-weighted MRI or pelvic examination.

Pelvic lymph node metastasis was assessed using abdominal computed tomography (CT) and/or ^18^F-fluorodeoxyglucose positron emission tomography/CT (FDG-PET/CT) and defined as a short-axis diameter >1.0 cm or maximum standardized uptake value >2.5.

### Outcome measures

Because the primary aim of this study was to develop a simple preoperative model to support treatment counseling, the primary outcome was receipt of postoperative adjuvant therapy, with treatment administration used as the reference outcome. To address the potential bias associated with using treatment administration as the primary outcome, secondary outcomes included (1) the presence of pathological indications for adjuvant therapy (parametrial invasion, pelvic lymph node metastasis, and Sedlis intermediate-risk criteria) and (2) the proportion of patients requiring postoperative adjuvant therapy across stratified categories of tumor size and SCC antigen levels

### Statistical analysis

The correlation between tumor size measured on MRI and preoperative serum SCC antigen levels was evaluated using Pearson’s correlation coefficient (*R*). The Cochran-Armitage trend test was used to evaluate trends across ordinal categories. Receiver operating characteristic (ROC) curve analysis was used to determine the optimal cut-off values using the Youden index (sensitivity + specificity − 1). Internal validation was performed using bootstrap resampling (1000 iterations), and the area under the curve (AUC) was used to assess model stability. Logistic regression analysis was performed to evaluate the association between dichotomized variables based on the proposed cut-off values and the presence of pathological indications for adjuvant therapy. Odds ratio (OR) and adjusted OR with 95% confidence intervals (CIs) were calculated. Survival was analyzed using the Kaplan–Meier method and compared using the log-rank test.

All analyses were performed using EZR (version 1.70), with a two-sided significance level of *P*<.05.^10^

## Results

**Figure 1:**
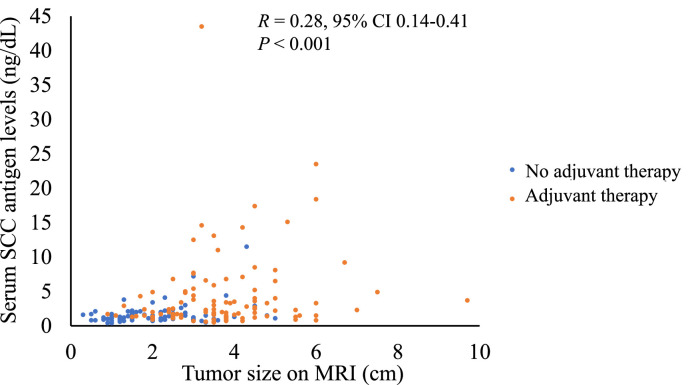
Scatter plot showing the correlation between tumor size on magnetic resonance imaging (MRI) and serum squamous cell carcinoma (SCC) antigen levels. A weak positive correlation was observed between tumor size measured on T2-weighted MRI and preoperative serum SCC antigen levels (*R*=0.28, 95% CI 0.14–0.41), *P*<.001. Each dot represents a single individual. Blue dots indicate patients who did not receive postoperative adjuvant therapy, and orange dots indicate those who did. The frequency of adjuvant therapy increased with tumor size and serum SCC antigen levels. Both variables showed a trend toward a higher frequency of postoperative adjuvant chemoradiotherapy with rising values.

This study included 191 patients with cervical SCC who underwent radical hysterectomy. The baseline characteristics are shown in Table 1. The median age, body mass index, tumor size, and serum SCC antigen levels were 43 years, 20.9 kg/m^2^, 3.0 cm, and 1.6 ng/mL, respectively. During follow-up,17 patients (8.9%) experienced recurrence and 7 (3.7%) died. Preoperative staging classified patients as IB1 (29.3%), IB2 (49.7%), and IB3 (21.0%). A weak positive correlation was observed between tumor size measured on MRI and serum SCC antigen levels (*R*=0.28, 95% CI 0.14–0.41, *P*< 0.001; Figure 1).

**Table 1:** Baseline characteristics of stage IB patients (*n*=191)

Patient characteristics at baseline	*n*=191
Age, y, median (range)	43 (28–76)
BMI, kg/m^2^, median (range)	20.9 (12.9–31.5)
Cervical conization	27 (14.1%)
Preoperative FIGO stage	
IB1, *n* (%)	56 (29.3%)
IB2, *n* (%)	95 (49.7%)
IB3, *n* (%)	40 (21.0%)
Tumor size on MRI, cm, median (range)	3.0 (0.3–9.7)
Serum SCC level, ng/mL, median (range)	1.6 (0.4–43.5)
Recurrence	17 (8.9%)
Death	7 (3.7%)

*BMI*, body mass index; *FIGO*, International Federation of Gynecology and Obstetrics; *SCC*, squamous cell carcinoma.

Maeda. Preoperative prediction of the need for postoperative adjuvant therapy in stage IB cervical cancer using tumor size measured. AJOG Glob Rep 2026.

The postoperative characteristics are shown in Table 2. Of the 191 patients, 104 (54.5%) received postoperative adjuvant therapy, including chemotherapy alone (1.6%), radiotherapy alone (14.1%), and concurrent CRT (37.2%). Based on pathological findings, 44 patients (23.0%) were classified as intermediate-risk and 60 (31.4%) as high-risk. Parametrial invasion, lymph node metastasis, and positive surgical margins were observed in 24 (12.6%), 47 (24.6%), and 3 patients (1.6%), respectively. Postoperative pathological staging showed that most patients had stage IB or IIIC1 disease, with detailed distributions provided in Table 2.

**Table 2:** Postoperative characteristics of stage IB patients (*n*=191)

Postoperative characteristics	*n*=191
Adjuvant therapy, *n* (%)	104 (54.5%)
Chemotherapy, *n* (%)	3 (1.6%)
Radiation therapy, *n* (%)	27 (14.1%)
Chemoradiotherapy, *n* (%)	74 (37.2%)
Intermediate risk, *n* (%)	44 (23.0%)
High risk, *n* (%)	60 (31.4%)
Parametrial invasion, *n* (%)	24 (12.6%)
Pelvic lymph node metastasis, *n* (%)	47 (24.6%)
Positive surgical margin, *n* (%)	3 (1.6%)
Final FIGO stage	
IB1	49 (25.7%)
IB2	62 (32.5%)
IB3	13 (6.8%)
IIA1	5 (2.6%)
IIA2	2 (1.0%)
IIB	13 (6.8%)
IIIC1	47 (24.6%)

*FIGO*, International Federation of Gynecology and Obstetrics.

**Figure 2:**
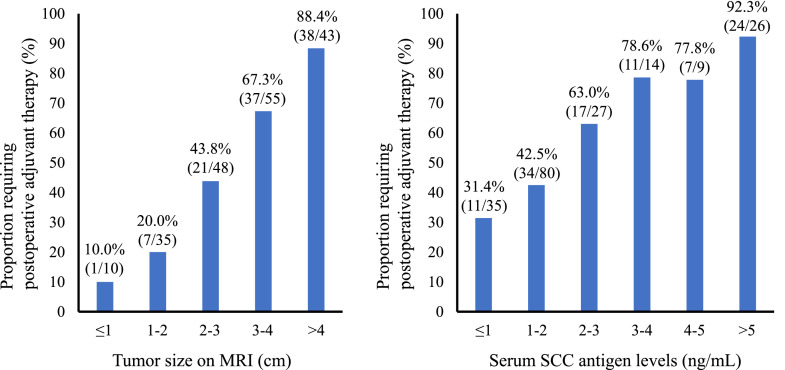
Proportion of patients requiring postoperative adjuvant therapy according to tumor size on magnetic resonance imaging (MRI) and serum squamous cell carcinoma (SCC) antigen level. The bar charts show the rates of postoperative adjuvant therapy stratified by (A) tumor size on MRI (≤1, 1–2, 2–3, 3–4cm, >4 cm), (B) preoperative serum SCC antigen level (≤1, 1–2, 2–3, 3–4, 4–5, >5 ng/mL).

First, the proportion of patients who received postoperative adjuvant therapy was investigated. When patients were stratified according to the tumor size measured on MRI and serum SCC antigen levels, the proportion of patients receiving adjuvant therapy increased significantly with increasing tumor size and SCC antigen levels (*P* for trend <.001 for both) (Figure 2, A and B).

Maeda. Preoperative prediction of the need for postoperative adjuvant therapy in stage IB cervical cancer using tumor size measured. AJOG Glob Rep 2026.

Next, to determine the cut-off values of tumor size measured on MRI and serum SCC antigen levels for predicting the need for adjuvant therapy in patients with stage IB cervical SCC, including analyses in both the overall cohort and the stage IB1 to IB2 cohort, ROC curve analysis was performed (Supplemental Figure 1, A–D). The optimal cut-off values for tumor size measured on MRI and serum SCC antigen levels were 3.2 cm and 2.3 ng/mL in the overall cohort, and 2.4 cm and 2.3 ng/mL in the stage IB1 to IB2 cohort. The AUC ROC for tumor size measured on MRI and serum SCC antigen levels were 0.82 (95% CI, 0.76–0.88) and 0.75 (95% CI 0.68–0.82) in the overall cohort, and 0.77 (95% CI, 0.69–0.84) and 0.73 (95% CI, 0.64–0.81) in stage IB1 to IB2 cohort, respectively. The combined model demonstrated robust performance, with AUCs of 0.85 (95% CI, 0.79–0.90) and 0.81 (95% CI, 0.75–0.88) in the overall and stage IB1 to IB2 cohorts, respectively (Supplemental Figure 1, E and F); bootstrap validation yielded similar AUCs of 0.85 (95% CI, 0.79–0.90) and 0.82 (95% CI, 0.75–0.88), indicating minimal overfitting.

**Figure 3:**
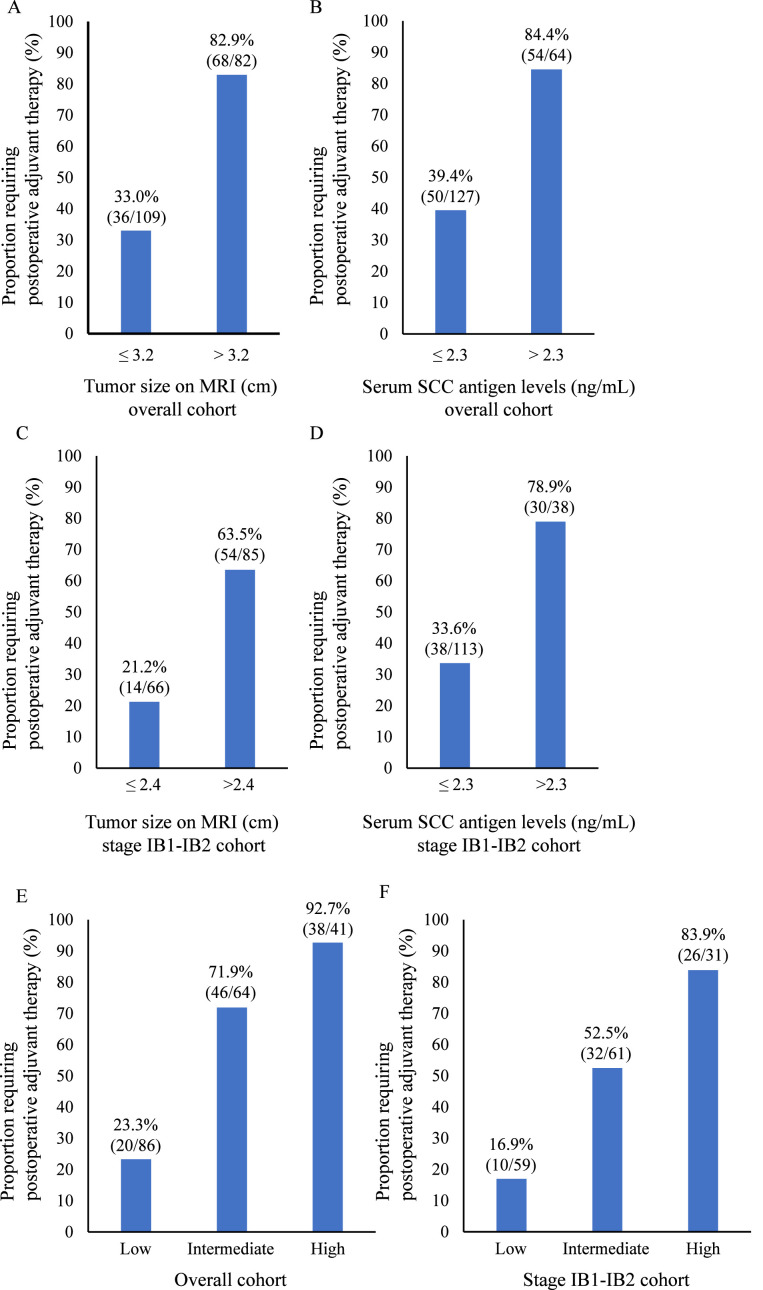
Proportion of patients requiring postoperative adjuvant therapy based on cutoff values of tumor size, serum squamous cell carcinoma (SCC) antigen level, and their combination. Bar graphs showing the proportion of patients who received postoperative adjuvant therapy, stratified by: (A) Tumor size measured on MRI (≤3.2 vs >3.2 cm) in the overall cohort; (B) preoperative serum SCC antigen level (≤2.3 vs >2.3 ng/mL) in the overall cohort; (C) tumor size measured on MRI (≤2.4 vs >2.4 cm) in the stage IB1 to IB2 cohort; (D) preoperative serum SCC antigen level (≤2.3 vs >2.3 ng/mL) in the stage IB1 to IB2 cohort; (E) number of predictive factors (0, 1, or 2) in the overall cohort; and (F) number of predictive factors (0, 1, or 2) in the stage IB1 to IB2 cohort. The predictive factors included tumor size above the cut-off value and SCC antigen level above the cut-off value. Patients were classified into three groups: low (0 factors), intermediate (1 factor), and high (2 factors). In the overall cohort, the respective proportions receiving adjuvant therapy were 23.3% (20/86), 71.9% (46/64), and 92.7% (38/41), demonstrating a significant increasing trend (*P* for trend <.001). In the stage IB1 to IB2 cohort, the corresponding proportions were 16.9% (10/59), 52.5% (32/61), and 83.9% (26/31), respectively (*P* for trend <.001).

Using these cutoff values, patients were dichotomized based on tumor size measured on MRI and serum SCC antigen levels. In the overall cohort, patients with tumor size >3.2 cm had a significantly higher rate of postoperative adjuvant therapy than those with ≤3.2 cm (82.9% vs 33.0%; OR 9.85, 95% CI 4.89–19.80; *P*<.001; Figure 3, A and Table 3). Similarly, elevated SCC antigen levels (>2.3 ng/mL) were associated with increased use of adjuvant therapy (84.4% vs 39.4%; OR 8.32, 95% CI 3.88–17.80; *P*<.001; Figure 3, B and Table 3). In the stage IB1 to IB2 cohort, similar associations were observed for tumor size (>2.4 cm: 63.5% vs 21.2%; OR 6.47, 95% CI 3.10–13.50; *P*<.001; Figure 3, C and Table 3) and SCC antigen levels (>2.3 ng/mL: 78.9% vs 33.6%; OR 7.40, 95% CI 3.09–17.70; *P*<.001; Figure 3, D and Table 3).

Maeda. Preoperative prediction of the need for postoperative adjuvant therapy in stage IB cervical cancer using tumor size measured. AJOG Glob Rep 2026.

**Table 3:** Uni- and multivariable logistic regression analyses of tumor size measured on MRI and serum SCC antigen levels for predicting the need for postoperative adjuvant therapy in the overall and stage IB1 to IB2 cohorts

	Univariable		multivariable	
	OR (95% CI)	*P*	aOR (95% CI)	*P*
Overall				
Size	9.85 (4.89–19.80)	<.001	8.02 (3.82–16.80)	<.001
SCC	8.32 (3.88–17.80)	<.001	6.41 (2.80–14.60)	.008
IB1–IB2				
Size	6.47 (3.10–13.50)	<.001	4.98 (2.30–10.80)	<.001
SCC	7.40 (3.09–17.70)	<.001	5.31 (2.11–13.30)	<.001

*aOR*, adjusted odds ratio; *CI*, confidence interval; *IB1–IB2*, stage IB1–IB2; *OR*, odds ratio; *SCC*, squamous cell carcinoma antigen; *Size*, tumor size.

In multivariable analysis, both tumor size and SCC antigen levels remained independent predictors in the overall cohort (tumor size: adjusted OR 8.02, 95% CI 3.82–16.80; SCC: adjusted OR 6.41, 95% CI 2.80–14.60; Table 3) and the stage IB1 to IB2 cohort (tumor size: adjusted OR 4.98, 95% CI 2.30–10.80; SCC: adjusted OR 5.31, 95% CI 2.11–13.30; Table 3) (all *P*<.001).

Then, Patients were stratified into three risk groups according to the number of predictive factors. The proportion of patients requiring postoperative adjuvant therapy increased significantly with increasing risk in both cohorts (*P* for trend <.001). In the overall cohort, adjuvant therapy rates were 23.3% (20/86), 71.9% (46/64), and 92.7% (38/41) in the low-, intermediate-, and high-risk groups (Figure 3, E), respectively. In the stage IB1 to IB2 cohort, the corresponding rates were 16.9% (10/59), 52.5% (32/61), and 83.9% (26/31) (Figure 3, F).

To address potential bias from using treatment administration as the primary outcome, we evaluated associations between the proposed cut-offs and pathological risk factors. In multivariable analysis, both tumor size and SCC antigen levels were independently associated with parametrial invasion (overall: adjusted OR 9.15 and 3.06), lymph node metastasis (adjusted OR 2.69 and 5.91), and high-risk features (adjusted OR 4.40 and 6.04), respectively, in the overall cohort. Similar associations were observed in the stage IB1 to IB2 cohort; however, tumor size was not significantly associated with parametrial invasion in this cohort. In contrast, neither variable was significantly associated with Sedlis criteria in the overall cohort, although tumor size showed a modest association in the IB1 to IB2 cohort (adjusted OR 2.45, *P*=.045) (Table 4).

**Table 4:** Uni- and multivariable logistic regression analyses of tumor size measured on MRI and serum SCC antigen levels for predicting pathological risk factors

			Univariable		Multivariable	
Outcome	Cohort	Variable	OR (95% CI)	*P*	aOR (95% CI)	*P*
PI	Overall	Size	12.20 (3.48–42.50)	<.001	9.15 (2.55–32.80)	<.001
		SCC	4.96 (1.99–12.30)	<.001	3.06 (1.17–8.04)	<.001
	IB1–IB2	Size	4.61 (1.60–13.30)	<.001	5.66 (0.67–47.90)	.11
		SCC	6.15 (1.69–22.40)	<.001	4.18 (1.1–15.80)	.035
LNM	Overall	Size	4.01 (1.99–8.10)	<.001	2.69 (1.26–5.76)	<.001
		SCC	7.47 (3.60–15.50)	<.001	5.91 (2.78–12.50)	<.001
	IB1–IB2	Size	6.46 (2.12–19.70)	.001	4.59 (1.45–14.50)	<.001
		SCC	5.39 (2.35–13.30)	<.001	3.96 (1.60–9.77)	<.001
High-risk	Overall	Size	5.97 (3.04–11.70)	<.001	4.40 (2.12–9.10)	<.001
		SCC	7.87 (3.96–15.60)	<.001	6.04 (2.93–12.50)	<.001
	IB1–IB2	Size	7.00 (2.54–19.30)	<.001	4.82 (1.67–13.90)	.003
		SCC	8.07 (3.49–18.70)	<.001	5.86 (2.44–14.10)	<.001
Int-risk	Overall	Size	1.90 (0.99–3.84)	.052	2.01 (0.99–4.10)	.054
		SCC	1.13 (0.56–2.27)	.74	0.90 (0.43–1.90)	.79
	IB1–IB2	Size	1.07 (2.49–5.81)	.034	2.45 (1.02–5.90)	.045
		SCC	1.40 (0.59–3.29)	<.001	1.06 (0.43–2.61)	.89

*aOR*, adjusted odds ratio; *CI*, confidence interval; *IB1–IB2*, stage IB1–IB2; *Int-risk*, intermediate risk; *LNM*, lymph node metastasis; *OR*, odds ratio; *PI*, parametrial invasion; *SCC*, squamous cell carcinoma antigen; *Size*, tumor size.

During follow-up, after a median follow-up period of 55 months, the 5-year recurrence-free survival rates were 92.6%, 91.2%, and 83.3% across risk groups (*P*=.36, log-rank test; Supplemental Figure 2, A), and the 5-year overall survival rates were 100%, 97.6%, and 80.4% (*P*<.001, log-rank test; Supplemental Figure 2, B). In the stage IB1 to IB2 cohort, recurrence occurred in 12 patients (7.9%), and 3 (2.0%) died, with corresponding 5-year recurrence-free survival rates of 92.8%, 86.5%, and 96.8% (*P*=.36, log-rank test) and overall survival rates of 100%, 100%, and 86.8% (*P*<.001, log-rank test; Supplemental Figure 2, C and D).

## Discussion

This retrospective study investigated the utility of tumor size measured on MRI and serum SCC antigen levels in predicting the need for adjuvant therapy in stage IB cervical SCC. The present study specifically focused on SCC, which represents the most common histological subtype of cervical cancer. Tumor size measured on MRI and serum SCC antigen levels were identified as predictive factors for receiving adjuvant therapy, and the combination of these two factors enhanced predictive accuracy both in the overall cohort and in the stage IB1 to IB2 cohort. The preoperative evaluation of these two factors is beneficial for treatment planning. Notably, when both risk factors were present, >90% of the patients in the overall cohort and >80% of the patients in the stage IB1 to IB2 cohort of patients required adjuvant therapy. In contrast, a lower proportion of patients without either factor required adjuvant therapy. These results support the use of preoperative imaging and serum biomarkers to determine the need for adjuvant therapy.

Our findings are consistent with and build upon those of previous research. Several studies have shown that the SCC antigen is associated with tumor size, parametrial invasion, and pelvic lymph node metastasis.^11^, ^12^, ^13^ In a previous study, preoperative SCC antigen levels exceeding 2.35 ng/mL could predict lymph node metastasis.^11^

Another study showed that SCC antigen is not merely a byproduct but actively contributes to tumor progression by promoting epithelial-mesenchymal transition, proliferation, and tumor invasion, providing a biological rationale for its association with aggressive disease.^14^ These findings provide a biological rationale for the strong clinical association between elevated serum SCC antigen levels and tumor expansion in cervical cancer.

Tumor size has also been shown to influence prognosis and treatment selection. The Sedlis criteria, which are widely used to determine intermediate risk, include tumor size as one of the three major components, along with deep stromal invasion and lymphovascular space invasion.^6^ While these are determined postoperatively, preoperative estimation of tumor size using MRI may be a valuable tool. Previous studies have demonstrated a strong correlation between MRI-measured and pathological tumor size, with reported correlation coefficients around 0.7 to 0.85 and concordance rates exceeding 80%.^15^, ^16^, ^17^ MRI has also been shown to have higher agreement with pathological findings than clinical examination or CT, supporting its reliability as a preoperative surrogate for tumor size.^15^ In previous studies, tumor size measured on MRI>2.5 cm has been associated with an increased risk of parametrial invasion, and a tumor size measured on MRI>2.9 cm has been associated with an increased risk of pelvic lymph node metastasis.^18^^,^^19^ In our study, a cut-off value of 3.2 cm in the overall cohort and 2.4 cm in the stage IB1 to IB2 cohort was identified to predict the need for adjuvant therapy. This is consistent with the observations of previous research and reinforces the clinical utility of tumor size measured on MRI as a predictor of advanced pathological features. When combined with serum SCC antigen levels, tumor size measured on MRI contributes to a robust preoperative risk stratification model.

A previous study reported that combining serum SCC antigen levels with MRI findings improves the accuracy of staging.^20^ The concept of integrating imaging findings with tumor markers in our study is consistent with this approach. However, few studies have evaluated the combined utility of serum SCC antigen levels and tumor size measured on MRI to predict the need for postoperative adjuvant therapy.

Our results demonstrate that tumor size measured on MRI and serum SCC antigen levels are independently associated with adverse pathological features, particularly lymph node metastasis and high-risk criteria, in both the overall and stage IB1 to IB2 cohorts. These findings support their utility as preoperative indicators of aggressive disease. In contrast, neither variable was strongly associated with Sedlis intermediate-risk criteria in the overall cohort, although tumor size showed a modest association in the IB1 to IB2 cohort, indicating limited predictive value for intermediate-risk features. Importantly, combining these variables improved predictive performance, and bootstrap validation confirmed stable model performance with minimal overfitting. Overall, our findings provide clinically applicable cut-off values and support the combined use of tumor size and SCC antigen levels as a practical tool for preoperative risk stratification and treatment decision-making. Radical hysterectomy or definitive chemoradiation for stage IB cervical cancer often depends on tumor size and patient factors such as age, comorbidities, fertility preservation, and the need for adjuvant therapy. Patients who require both surgery and postoperative radiation or CRT are at an increased risk of treatment-related morbidity.^5^^,^^7^^,^^8^ Therefore, preoperative identification of patients who are likely to require adjuvant therapy may help inform initial treatment planning and potentially reduce the risk of dual-modality treatment. Furthermore, for younger patients concerned about ovarian preservation or fertility-sparing strategies, accurate preoperative risk assessment may support decision-making. These results suggest that the tumor size measured on MRI and serum SCC antigen levels can be useful for preoperative counseling and multidisciplinary planning.

Our study has various strengths. First, a homogeneous cohort of patients with stage IB cervical SCC treated with type C1 radical hysterectomy was included, excluding those with known parametrial invasion or preoperative lymph node metastasis. These strengths enhance the usefulness of these findings in surgically eligible patients with stage IB disease. Second, readily available parameters such as tumor size measured on MRI and serum SCC antigen levels were used. Third, the patients were stratified into three groups (low-, intermediate-, and high-risk) based on the number of predictive factors. This approach is simple and easily applicable in clinical settings.

This study has a few limitations. First, it was conducted at a single institution with a relatively small number of patients. Second, this was a single-center study conducted in a Japanese population, which may limit the external validity of our findings. In particular, the relative ethnic homogeneity of the study population may influence the generalizability of the proposed cut-off values, as tumor biology, body habitus, and baseline SCC antigen levels may vary across different populations. Furthermore, our definition of adjuvant therapy included both intermediate- and high-risk patients. Although these findings reflect routine clinical practice, the indications for adjuvant therapy may vary. Third, the cut-off values for tumor size measured on MRI and serum SCC antigen levels were derived based on the receipt of postoperative adjuvant therapy, which may be influenced by physician discretion and institutional practice rather than purely objective disease biology. To mitigate this potential bias, we additionally evaluated the association between these variables and established pathological risk factors, including parametrial invasion, lymph node metastasis, and high-risk criteria. The consistent associations observed in these analyses support the biological relevance and robustness of our findings. Fourth, this study did not include potential pathological variables such as lymphovascular space invasion and stromal invasion, as they cannot be reliably evaluated before surgery. Instead, we focused on preoperative parameters that can be easily and objectively assessed.

This study suggests potential implications for both clinical practice and future research. Tumor size on MRI and serum SCC antigen levels are readily evaluated in the preoperative setting at most institutions. The present findings indicate that these parameters may be useful for stratifying patients according to their likelihood of requiring postoperative adjuvant therapy, thereby supporting more individualized treatment planning. Nevertheless, given the retrospective nature of this study, prospective multicenter investigations are necessary to validate these observations. Future studies should also assess the reproducibility and clinical applicability of the proposed cut-off values for tumor size measured on MRI and serum SCC antigen levels.

## Conclusion

This study demonstrated that preoperative tumor size measured on MRI and serum SCC antigen levels are predictors of the need for postoperative adjuvant therapy in patients with stage IB cervical SCC. These parameters may facilitate the identification of patients with a high probability of exhibiting pathological risk features, thereby guiding preoperative counseling and multidisciplinary treatment planning.

## Abbreviations

CRT—Chemoradiotherapy

MRI—Magnetic Resonance Imaging

SCC—Squamous Cell Carcinoma

FIGO—International Federation of Gynecology and Obstetrics

CT—Computed Tomography

FDG-PET/CT—18F-fluorodeoxyglucose Positron Emission Tomography/Computed Tomography

ROC—Receiver Operating Characteristic

AUC—Area Under the Curve

OR—Odds ratio

## CRediT authorship contribution statement

**Michihide Maeda:** Writing – review & editing, Writing – original draft, Methodology, Formal analysis, Conceptualization. **Miho Kitai:** Methodology, Investigation, Conceptualization. **Masashi Akada:** Data curation. **Yusaku Shimizu:** Data curation. **Eri Yamabe:** Data curation. **Reisa Kakubari:** Data curation. **Shinya Matsuzaki:** Writing – original draft, Investigation. **Tsuyoshi Hisa:** Investigation. **Shinsuke Koyama:** Methodology. **Seiji Mabuchi:** Writing – review & editing, Supervision.

## Appendix Supplementary materials

Image, application 1

Image, application 2

Image, application 3

## Footnotes

Supplementary material associated with this article can be found in the online version at doi:10.1016/j.xagr.2026.100671.

## References

[1] Bray F., Laversanne M., Sung H. (2024). Global cancer statistics 2022: GLOBOCAN estimates of incidence and mortality worldwide for 36 cancers in 185 countries. CA Cancer J Clin.

[2] Bhatla N., Aoki D., Sharma D.N. (2021). Cancer of the cervix uteri: 2021 update. Int J Gynaecol Obstet.

[3] Abu-Rustum N.R., Yashar C.M., Arend R. (2023). NCCN Guidelines® insights: cervical cancer, version 1.2024. J Natl Compr Canc Netw.

[4] Eighamrawi K.A., Haggag M.H., Habib EE. (2011). Treatment complications among long-term survivors of cervical cancer: treated by surgery or radiotherapy. Oncol Rev.

[5] Monk B.J., Wang J., Im S. (2005). Rethinking the use of radiation and chemotherapy after radical hysterectomy: a clinical-pathologic analysis of a Gynecologic Oncology Group/Southwest Oncology Group/Radiation Therapy Oncology Group trial. Gynecol Oncol.

[6] Sedlis A., Bundy B.N., Rotman M.Z. (1999). A randomized trial of pelvic radiation therapy versus no further therapy in selected patients with stage IB carcinoma of the cervix after radical hysterectomy and pelvic lymphadenectomy: a Gynecologic Oncology Group Study. Gynecol Oncol.

[7] Yamashita H., Okuma K., Kawana K. (2010). Comparison between conventional surgery plus postoperative adjuvant radiotherapy and concurrent chemoradiation for FIGO stage IIB cervical carcinoma: a retrospective study. Am J Clin Oncol.

[8] Kim J.H., Choi J.H., Ki E.Y. (2012). Incidence and risk factors of lower-extremity lymphedema after radical surgery with or without adjuvant radiotherapy in patients with FIGO stage I to stage IIA cervical cancer. Int J Gynecol Cancer.

[9] Peters W.A., Liu P.Y., Barrett R.J. (2000). Concurrent chemotherapy and pelvic radiation therapy compared with pelvic radiation therapy alone as adjuvant therapy after radical surgery in high-risk early-stage cancer of the cervix. J Clin Oncol.

[10] Kanda Y. (2013). Investigation of the freely-available easy-to-use software “EZR” (Easy R) for medical statistics. Bone Marrow Transplant.

[11] Xu D., Wang D., Wang S. (2017). Correlation between squamous cell carcinoma antigen level and the clinicopathological features of early-stage cervical squamous cell carcinoma and the predictive value of squamous cell carcinoma antigen combined with computed tomography scan for lymph node metastasis. Int J Gynecol Cancer.

[12] Reesink-Peters N., van der Velden J., Ten Hoor K.A. (2005). Preoperative serum squamous cell carcinoma antigen levels in clinical decision making for patients with early-stage cervical cancer. J Clin Oncol.

[13] Zhu C., Zhang W., Wang X. (2021). Predictive value of preoperative serum squamous cell carcinoma antigen level for lymph node metastasis in early-stage cervical squamous cell carcinoma. Medicine.

[14] Chen L., Shi V., Wang S. (2023). SCCA1/SERPINB3 suppresses antitumor immunity and blunts therapy-induced T cell responses via STAT-dependent chemokine production. J Clin Invest.

[15] Salvo G., Gonzalez Martin A., Gonzales N.R. (2020). Role of imaging in pretreatment evaluation of early-stage cervical cancer: comparison of MRI, CT, and clinical examination. Int J Gynecol Cancer.

[16] Pan T.L., Pareja R., Chiva L. (2024). Accuracy of pre-operative tumor size assessment compared to final pathology and frequency of adjuvant treatment in patients with FIGO 2018 stage IB2 cervical cancer. Int J Gynecol Cancer.

[17] Jung D.C., Ju W., Choi H.J. (2008). The validity of tumour diameter assessed by magnetic resonance imaging and gross specimen with regard to tumour volume in cervical cancer patients. Eur J Cancer.

[18] He F., Du J., Chen X. (2018). Assessment of parametrial involvement in early stages cervical cancer with preoperative magnetic resonance imaging. Int J Gynaecol Cancer.

[19] Chen X.L., Chen G.W., Xu G.H. (2018). Tumor size at magnetic resonance imaging association with lymph node metastasis and lymphovascular space invasion in resectable cervical cancer: a multicenter evaluation of surgical specimens. Int J Gynaecol Cancer.

[20] Ran C., Sun J., Qu Y. (2021). Clinical value of MRI, serum SCCA, and CA125 levels in the diagnosis of lymph node metastasis and para-uterine infiltration in cervical cancer. World J Surg Onc.

