# Supplementary material for: Preoperative prediction of the need for postoperative adjuvant therapy in stage IB cervical cancer using tumor size measured on magnetic resonance imaging and serum squamous cell carcinoma antigen levels
Source: AJOG Glob Rep. 2026 Jun 28;6(3):100671. doi: 10.1016/j.xagr.2026.100671 (PMC13427441; doi:10.1016/j.xagr.2026.100671)
Supplement: Supplementary file 1 [file mmc1.docx]

**Supplementary Material**

Supplementary figure 1. Receiver operating characteristic (ROC) curves for predicting the need for postoperative adjuvant therapy using tumor size on magnetic resonance imaging (MRI) and serum squamous cell carcinoma antigen levels.

(A) ROC curve of tumor size measured on MRI for predicting the need for postoperative adjuvant therapy in the overall cohort. (B) ROC curve of serum SCCantigen levels for predicting the need for postoperative adjuvant therapy in the overall cohort. (C) ROC curve of tumor size measured on MRI for predicting the need for postoperative adjuvant therapy in the stage IB1-IB2 cohort. (D) ROC curve of serum SCC antigen levels for predicting the need for postoperative adjuvant therapy in the stage IB1-IB2 cohort. (E) ROC curve of the combined model for predicting the need for postoperative adjuvant therapy in the overall cohort. (F) ROC curve of the combined model for predicting the need for postoperative adjuvant therapy in the stage IB1-IB2 cohort. Black dots indicate the cutoff values. Data are presented as cut-off values (sensitivity and specificity). AUC, area under the curve.

Supplementary Figure 2. Kaplan-Meier curves according to risk classification.

(A) Recurrence-free survival in the overall cohort. (B) Overall survival in the overall cohort. (C) Recurrence-free survival in the stage IB1-IB2 cohort. (D) Overall survival in the stage IB1-IB2 cohort. The numbers at risk are shown per group at the bottom of each figure.
