# Supplementary figures and images for: Preoperative prediction of the need for postoperative adjuvant therapy in stage IB cervical cancer using tumor size measured on magnetic resonance imaging and serum squamous cell carcinoma antigen levels
Source: AJOG Glob Rep. 2026 Jun 28;6(3):100671. doi: 10.1016/j.xagr.2026.100671 (PMC13427441; doi:10.1016/j.xagr.2026.100671)

Supplementary Figure 1

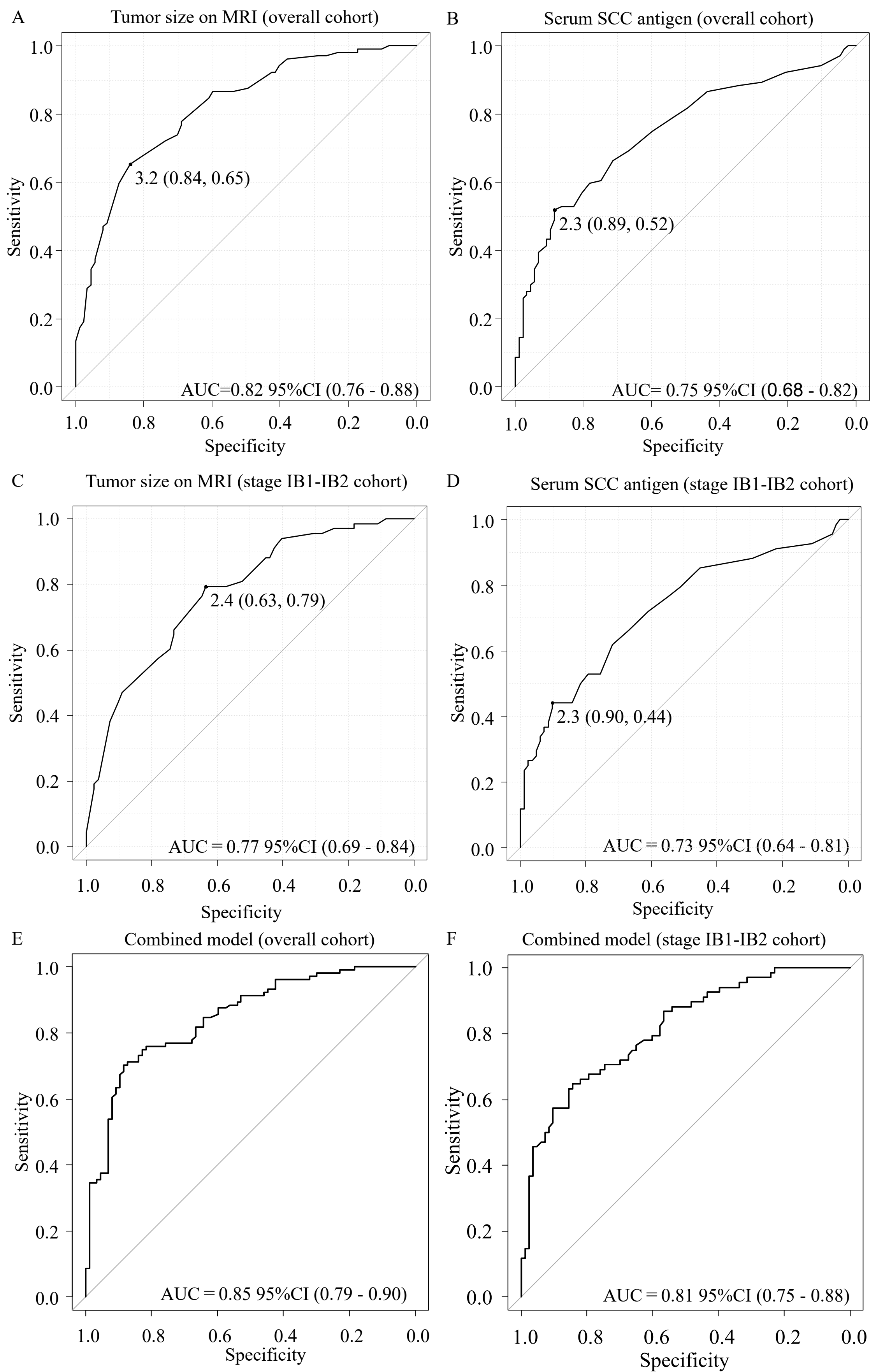

Supplement: Supplementary file 2 [file mmc2.pdf]

Supplementary Figure 2

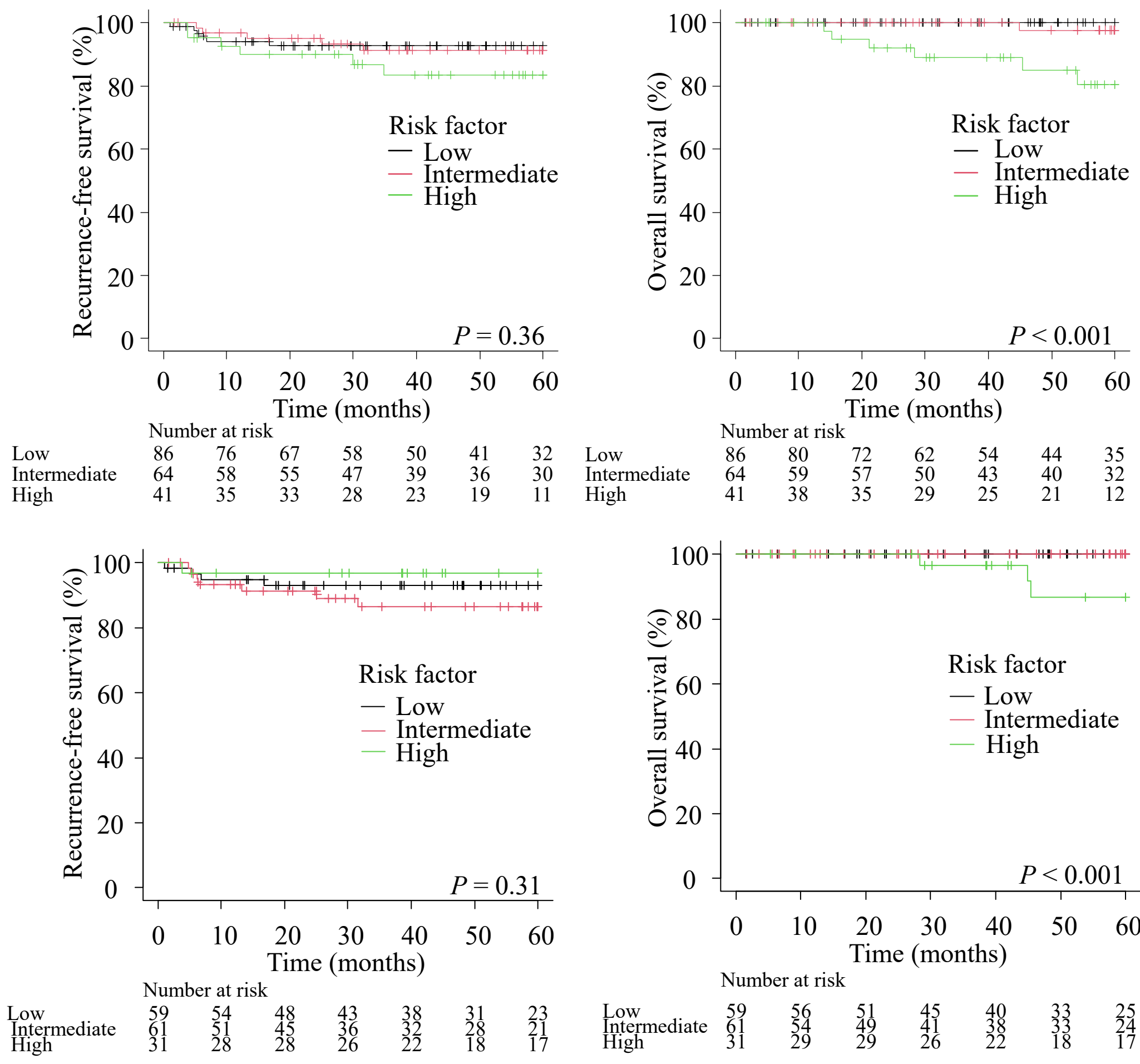

Supplement: Supplementary file 3 [file mmc3.pdf]
